# The Reliability of Kaplan’s Cardinal Line as a Potential Surface Marker for the Superficial Palmar Arch During Carpal Tunnel Release: An Anatomical Study With Surgical Perspective

**DOI:** 10.7759/cureus.35144

**Published:** 2023-02-18

**Authors:** Harsimarjit Kaur, Apurba Patra, Manjeet Singh, Gurdeep S Kalyan, Adil Asghar

**Affiliations:** 1 Anatomy, Government Medical College Patiala, Patiala, IND; 2 Anatomy, All India Institute of Medical Sciences, Bathinda, Bathinda, IND; 3 Orthopedics, Maharishi Markandeshwar Institute of Medical Sciences and Research, Ambala, IND; 4 Anatomy/Orthopedics, All India Institute of Medical Sciences, Patna, Patna, IND

**Keywords:** superficial palmar arch, topography, hand surgery, carpal tunnel, anatomical landmark

## Abstract

Purpose

Kaplan's cardinal line (KCL) provides a more accurate reference point to the superficial palmar arch (SPA). The aim was to determine the KCL-SPA distances and their relationship with the other defined superficial landmarks, such as distal wrist crease (DWC) or distal portion of the transverse carpal ligament (TCL) or DWC-TCL distance. The objective was to determine the distal limit of the incision made during carpal tunnel release (CTR).

Methods

Sixty hands were dissected after KCL was drawn on each hand using standard methods. The distance from KCL to the SPA was measured along the radial and ulnar borders of the ring finger and recorded as radial and ulnar KCL-SPA distance, respectively. The distance between the DWC and the distal portion of the TCL was also measured (DWC-TCL). Correlation analysis was done between the DWC-TCL and KCL-SPA distance. The ratios between the radial and ulnar KCL-SPA distance and DWC-TCL distance were calculated and mentioned as radial and ulnar Kaplan cardinal index, respectively.

Results

KCL-SPA distance was 6.8±3.7 mm along the radial border and 6.6±3.6 mm along the ulnar border of the ring finger. The DWC-TCL distance was 29.4±1.2 mm. The means of radial and ulnar Kaplan cardinal indices were 0.23 and 0.22, respectively. A significant correlation was found between the DWC-TCL distance and the KCL-SPA distances.

Conclusion

Clinically, KCL can be appraised as a predictable surface landmark in limiting the distal-most extent of the incision during CTR and protecting SPA from transection. The SPA was found to lie at a variable distance from the KCL, and the minimum distance was found to be 3.3 mm. This should be considered as the maximum permissible extension of CTR incision beyond KCL.

## Introduction

Clinicians, especially surgeons, use surface marking as an important tool to make out a picture of underlying structures to avoid undue complications during procedures. In hand surgery, carpal tunnel release (CTR) is one of the commonly performed outpatient procedures both by open and endoscopic approach. Although CTR is comparatively a simple and superficial procedure, the chances of fatal complications, such as inadvertent injury to the superficial palmar arch (SPA), are not uncommon. SPA, a superficial continuation of the ulnar artery, runs transversely from the ulnar side to the radial side to join with its radial counterpart and make the arch complete. Variability in its location and perpendicular position to the line of incision in CTR makes it vulnerable to cut [1]. So, the preciseness of the incision, especially the distal limit of the incision, is the key step in preventing iatrogenic injury and establishing a desirable outcome. The line of incisions and their distal extension can be planned considering the potential areas of neurovascular vulnerability. The ongoing debate in the literature is about the most vulnerable structure during CTR being the SPA, the distal extent of the ulnar artery [2]. Understanding the relationship between overlying surface markers and variability of the SPA might help in determining "safe zones" for CTR incisions. Kaplan's cardinal line (KCL), originally described in 1953 by E. B. Kaplan [3], is described as a line "drawn from the apex of the inter-digital fold between the thumb and index finger toward the ulnar side of the hand, parallel with the middle crease of the hand." Previous researchers agreed that incisions for both open and endoscopic CTR should be limited distally by the intersection of a longitudinal incision with the original KCL (OKCL) to prevent inadvertent injury to the SPA [4, 5]. However, anatomical variations in KCL or other landmarks make the job cumbersome [6-10]. Additionally, according to current surgical textbooks, KCL represents an accurate surface marker for the deep palmar arterial arch instead of superficial [3], even though they differ in the portrayal of KCL [11]. Hence, such landmarks should be precise and reliable to ensure relatively safe zones for surgical incisions. To date, the original description of KCL represents a surface marker for the clinical take-off point of the SPA. In performing both open and endoscopic CTR, the distal extent of an incision placed at the intersection of KCL and the line along the long axis of the ring finger is a point past which iatrogenic injury to the SPA is possible [12, 13]. Hence, it becomes paramount to accurately define the landmark and its relationship with other defined superficial landmarks such as distal wrist crease (DWC) or distal portion of the transverse carpal ligament (TCL). With such a background, the primary aim of the study was to determine the KCL-SPA distances and their relationship with DWC-TCL distance. The objective was to determine the distal limit of the incision made during CTR and, thus, the clinical applicability of KCL as a predictable surface marker. This article was previously presented as a meeting abstract at FIRMST International Conference on Applied Anatomy from 11 to 12 February 2022 (FICAA 2022).

## Materials and methods

**Figure 1:**
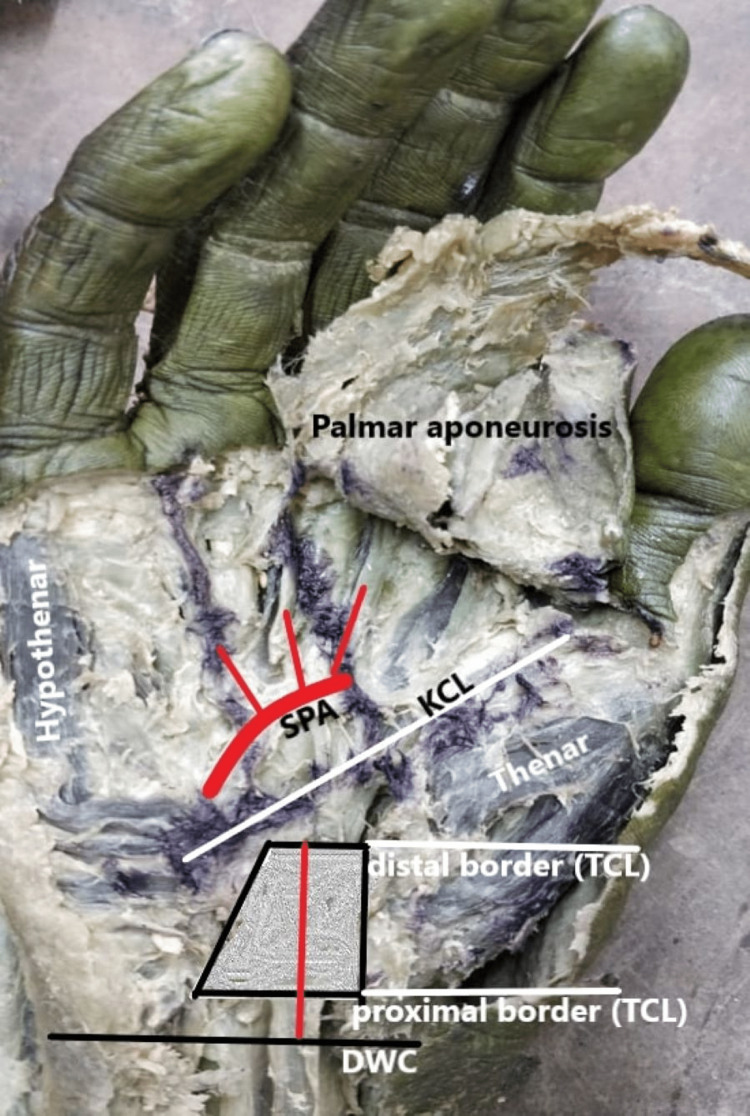
Measurement of DWC-TCL distance: red line extending from the midpoint of the distal wrist crease (DWC) to the midpoint of the distal border of TCL of the right hand. SPA: Superficial palmar arch; KCL: Kaplan’s cardinal line; TCL: Transverse carpal ligament.

Thirty adults (23 male, seven female) embalmed cadavers (60 hands) aged between 45 and 65 years were dissected in accordance with the Declaration of Helsinki and were accepted by the Ethics Committee of Government Medical College, Patiala, India (reference No. Trg9(/310/2020). Based on the availability of the human cadavers in our dissection laboratory, we used 30 cadavers (60 hands) as a convenience sampling. The embalming was done with a basic suspension of formalin (10%) (the actual amount of formaldehyde dissolved in 10% formalin is only 3.7-4.0%) to achieve complete and uniform fixation with minimum tissue shrinkage. Cadavers with a history of previous hand surgery, deformity or damage of the upper extremity, history of musculoskeletal diseases, nervous diseases, or connective tissue disorders were excluded from the study. Surface anatomical markers of the palm (DWC, palmar creases, and first web space) were identified first. Subsequently, the classical KCL was marked on the palm with methylene blue as per the classical description of Kaplan [3], along with the two longitudinal lines extending from the radial and ulnar borders of the ring finger. Palmar skin was incised along the lines of marking, and skin flaps were removed to reveal the palmar aponeurosis, which was then reflected distally, exposing the TCL. The TCL was recognized by its transversely disposed fibers, running from the tubercle of the scaphoid and the trapezium on the radial side to the pisiform and the hook of the hamate bone on the ulnar side. Careful dissection was done to identify the ulnar artery at the level of Guyon's canal and traced distally towards its continuation as SPA. Any variations in SPA in the form of the complete or incomplete arch were observed and noted. The distances between the DWC and TCL (distal margin) were measured and recorded as DWC-TCL distances (Figure 1).

**Figure 2:**
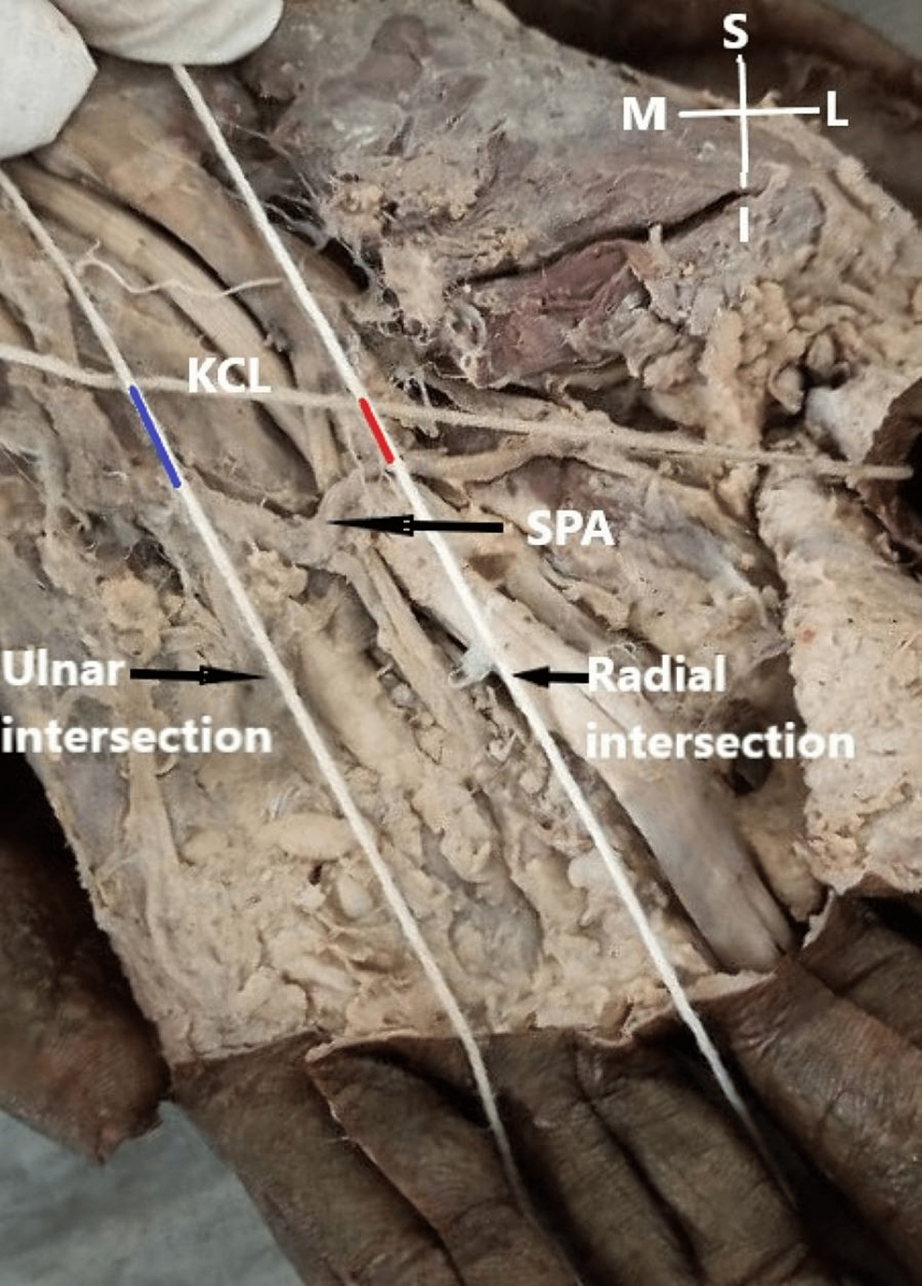
Kaplan's cardinal line, radial and ulnar longitudinal intersection extending from radial and ulnar borders of the ring finger were represented on the dissected hand (left) with the help of silk thread. Red line: Distance between the radial border ring finger and superficial palmar arch; Blue line: Distance between ulnar border ring finger and superficial palmar arch. M: Medial; L: Lateral; S: Superior; I: Inferior.

Simultaneously, the points of intersection of the longitudinal lines with KCL were marked. The distances from the intersection of the KCL with the radial and ulnar borders of the ring finger proximally to the SPA distally were measured and recorded as radial and ulnar KCL-SPA distances, respectively (Figure 2).

A single researcher took all the measurements with the help of high-precision digital vernier calipers (Mitutoyo Corp, Japan) with the least count of 0.01 mm. The same observer took three different readings at different points in time, and their mean was considered the final for data analysis. The ratios between the radial and ulnar KCL-SPA distance and DWC-TCL were calculated and mentioned as radial and ulnar Kaplan cardinal index (KI), respectively.

Statistical analysis

Data obtained were recorded as mean ± SD in the Microsoft Excel sheet. Statistical analysis was performed using descriptive statistics SPSS version 20.0 software for windows 10. The measured parameters were compared using an unpaired t-test (p-value < 0.05 was considered statistically significant). Pearson correlation analysis was performed between each limb's DWC-TCL and KCL-SPA distance.

## Results

**Figure 3:**
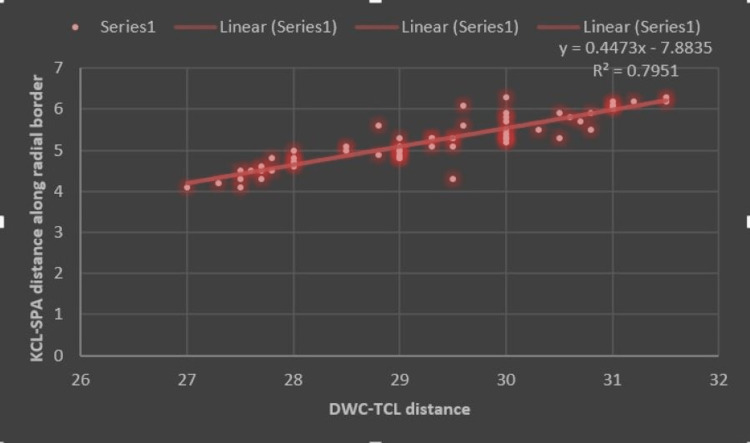
Scattered plot showing the relationship between KCL-SPA (R) distance along the radial border of ring finger and DWC-TCL length. KCL-SPA: Kaplan's cardinal line-Superficial palmar arch; DWC-TCL: Distal wrist crease-transverse carpal ligament.

**Figure 4:**
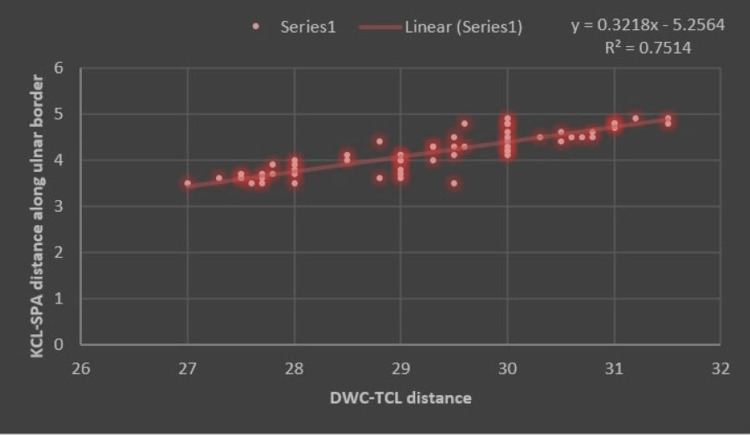
Scattered plot showing the relationship between KCL-SPA (U) distance along the ulnar border of the ring finger and DWC-TCL length. KCL-SPA: Kaplan's cardinal line-Superficial palmar arch; DWC-TCL: Distal wrist crease-transverse carpal ligament.

Various configurations of the SPA were noted during dissections. Complete arches were reported in 47/60 hands (78.3%). The radial artery was the main secondary contributor to completing the SPA in all cases with a complete arch. The KCL-SPA distance was 6.8±3.7 mm (ranges between 3.6 mm and 13.7 mm) along the radial border (R) and 6.6±3.6 mm (ranges between 3.3 mm and 11.2 mm) along the ulnar border (U) of the ring finger. The mean DWC-TCL distance was 29.4 ± 1.2 mm. The means of radial and ulnar KIs were 0.23 (ranges between 0.12 and 0.46) and 0.22 (ranges between 0.11 and 0.38), respectively. Measured parameters showed no significant difference between the sides. However, a significant correlation was found between the DWC-TCL distance and the KCL-SPA distances (Figures 3-4).

## Discussion

Our findings showed a significant correlation between both the radial and ulnar KCL-SPA distances and the DWC-TCL distance. Previously also, few researchers have deduced the average KCL-SPA distance in various populations and thus derived the safe distance for CTR incision. However, this so-called 'safe distance' varies widely according to different authors (Table 1).

**Table 1:** The average KCL-SPA distances reported by various authors. KCL: Kaplan’s cardinal line; SPA: Superficial palmar arch.

Authors	Year of study	Sample size (cadaveric hands)	Average KCL-SPA distance (in mm)
Lee WP and Strickland JW [13]	1998	28	10.0
Vasiliadis HS, et al. [14]	2006	16	10.4 ± 5.0
Vella JC, et al. [15]	2006	-	14.0 ± 5.0
McLean KM, et al. [5]	2008	48	15.3 ± 8.6
Panchal AP and Trzeciak MA [16]	2010	60	11.8 ± 4.0
Varalakshmi KL [17]	2021	40	8.3 ± 1.0
Present study	2022	60	6.8 ± 3.7

When comparing our findings with the previous authors, the KCL-SPA distance we reported was lesser than theirs. Previous literature showed (Table 1) that average KCL-SPA distances range between 8.32 and 15.3 mm, theoretically allowing some inherent flexibility in the planning of the incision for open and endoscopic CTR [2, 15]. On the contrary, we found SPA much closer to the KCL, making the structure more prone to inadvertent injury during open or endoscopic CTR. Lee WP and Strickland JW [13] examined the reliability of the KCL as the distal-most extent of the incision in limited incision technique. They found KCL 10 mm proximal to the SPA, whereas we reported this distance to be roughly 7 mm. Hence, in a limited incision technique, if the cutting tome is placed on the KCL distally and extended proximally, complete ligament release can be obtained without any neurovascular jeopardy. Thus, our findings align with the notion put in place by Lee WP and Strickland JW [13]. Reviewing previous literature leaves us with much uncertainty as the 'safe distance' of SPA from KCL varies widely according to the different authors. This can be due to the differences in individual diagnostic criteria, measuring techniques, observer error, and confounding factors of the population or ethnic group under study. Most authors [5, 13-15] have measured the KCL-SPA distance as a vertical length between the midpoint of the KCL line and the midpoint of SPA. Whereas, as in the present study, we have taken the KCL-SPA distance both along the radial and ulnar border of the ring finger. Moreover, we placed silk threads along the medial and ulnar border of the ring finger and measured the length of the thread as KCL-SPA distance. In comparison, few authors have measured the distance directly with the vernier calipers. Most importantly, if we think about the noted difference in findings between ours and previous studies, it is because most of the previous studies were done on fresh frozen cadavers, while ours was on formalin-preserved hands. Hence, tissue shrinkage caused by formalin might be the factor behind decreased KCL-SPA distance in our study. Most of the authors mentioned in Table 1 have measured the vertical KCL-SPA distance and advocated using the same as the maximum permissible length of CTR incision. However, presently we did not find SPA constantly parallel to the KCL, and the position of the artery varies at different points along its course. Hence, considering KCL-SPA distance as the safe length for CTR incision may still result in some amount of SPA injury. Moreover, as the distance of the artery from the KCL varies at different points along its course, it is better to take the shortest distance as the maximum permissible extension of CTR to minimize the risk of SPA damage.

Previously, most of the authors, even though they have derived the safe distance, have not correlated the derived value with other widely used defined anatomical landmarks as morphometric variables, more precisely with DWC and distal border of TCL distance. In the present work, we reported a strong positive correlation between KCL-SPA distance and DWC-TCL distance. There was a strong positive correlation (R=0.79) between the KCL-SPA distance along the radial border of the ring finger and the DWC-TCL distance. Since there was a positive linear correlation, the KCL-SPA distance or the safe extension of CTR incision can be predicted more precisely for a given value of DWC-TCL distance using the linear regression equation. R2 value which determines the variation in the KCL-SPA distance (R) and can be attributed to variation in the DWC-TCL length, was almost 80%. Such a very high value determines the reliability of KCL as an acceptable anatomical landmark for hand surgery, more precisely, CTR. Similar trends were also seen between the KCL-SPA distance along the ulnar border (U) of the ring finger and DWC-TCL length, though R2 was 0.75. Hence, the predictability of the KCL-SPA distance along the radial border of the ring finger was slightly better than that of the KCL-SPA distance along the ulnar border. To date, no studies have reported such a correlation that defines the authenticity and reliability of KCL as a surface landmark during CTR. Additionally, we have computed the Kaplan cardinal indices, which range from 0.11 to 0.46. The lesser the value of KI, the larger the length of the safe distance, thereby allowing inherent flexibility in the planning of the incision for open and endoscopic CTR. To date, KCL is considered the most reliable surface landmark to localize important anatomical structures such as SPA [18]. Additionally, other related surface markers such as fat pad surrounding the SPA, alternate version of KCL, and DWC may be considered for better surgical outcomes. In addition to the KCL, Vasiliadis HS et al. [14] also used a pad of fat surrounding the SPA as a useful guide to protect the SPA during distal ligament release. Tsuruta T et al. [12] and Rotman MB and Manske PR [8] used the fat pad as a surface marker but reported difficulty visualizing the SPA. These studies showed that KCL is more reliable than fat pad signs. Also, using the fat pad in combination with the previous one does not improve surgical outcomes.

Vella JC et al. [15] also utilized an alternate version of KCL based on Kaplan's subsequent description in 1968 [19] and reported an average distance of 11±4 mm. While this line is apparently distal to the OKCL and safely away from the SPA, this alternate version of OKCL is less reliable, as a history of trauma to the pisiform may alter its shape or location. Moreover, in obese individuals, palpation of the exact boundaries of pisiform can be difficult. Additionally, McLean KM et al. [5], Varalakshmi KL et al. [17], and Ozcanli H et al. [20] have utilized DWC to locate and protect SPA during CTR. The average distance of SPA from DWC was 44.2 ± 4.9 mm, 46 ± 3.9 mm, and 48 mm, respectively. Presently, we have not considered the DWC as a potential surface marker but utilized it to compute the DWC-TCL distance and used this distance as a potential predictor of KCL-SPA distance or safe distance for CTR. Utilizing a consistent surface marker such as KCL as a standard reference line may help to define the distal extent of the incision and may help to plan complication-free CTR [21]. However, adequate surgical training and cadaveric workshops on hand surgery are more crucial as CTR, specifically endoscopic CTR, poses a high level of technical difficulty. There exist some limitations to the present study. Most importantly, the study was done on cadaveric hands preserved with formaldehyde, which causes tissue shrinkage and might have altered the vascular relationships. Thus, living or in vivo conditions could be different. We have studied only the interrelationship between KCL and SPA and their relationship with mid-hand length, thus not taking other factors (surface markers such as fat pad surrounding the SPA and the alternate version of KCL and DWC) into consideration, which may affect the surgical outcome. The sample size was small; moreover, the number of female cadavers restricted us from comparing the values.

## Conclusions

In conclusion, KCL can be appraised as a predictable surface landmark in limiting the distal-most extent of the incision during CTR and protecting SPA from transection. The SPA was found to lie at a variable distance from the KCL. The minimum KCL-SPA distance was 3.3 mm, which should be considered the maximum permissible extension of CTR incision beyond KCL. DWC-TCL distance used this distance as a potential predictor of KCL-SPA distance or safe distance for CTR. Utilizing a consistent surface marker such as KCL as a standard reference line may help to define the distal extent of the incision and may help to plan complication-free CTR. However, adequate surgical training and cadaveric workshops on hand surgery are more crucial as CTR, specifically endoscopic CTR, poses a high level of technical difficulty.
